# Effect of paddy straw plus nonforage fiber sources based complete rations with different levels of neutral detergent fiber on hemato-biochemical and mineral profile of lactating dairy cows

**DOI:** 10.14202/vetworld.2017.836-842

**Published:** 2017-07-29

**Authors:** Biju Chacko, K. M. Syam Mohan, K. Ally, K. Shyama, K. S. Anil, C. T. Sathian

**Affiliations:** 1 Department of Animal Nutrition, College of Veterinary and Animal Sciences, Mannuthy - 680 651, Thrissur, Kerala, India; 2 University Livestock Farm & Fodder Research and Development Scheme, Mannuthy - 680 651, Thrissur, Kerala, India; 3 Department of Dairy Science, College of Veterinary and Animal Sciences, Mannuthy - 680 651, Thrissur, Kerala, India

**Keywords:** blood urea nitrogen, complete feeds, cows, hematology, neutral detergent fiber, paddy straw, plasma

## Abstract

**Aim:**

This study was conducted to assess the effect of feeding paddy straw plus nonforage fiber sources based complete rations with different levels of neutral detergent fiber (NDF) on hemato-biochemical and mineral parameters of lactating dairy cows.

**Materials and Methods:**

The study was conducted for 6 months in 18 lactating dairy cows, divided into three groups of six each, by feeding them on paddy straw plus nonforage fiber sources based complete rations containing different levels of NDF, in two phases of 3 months (90 days) each, being the early and mid lactation phases, respectively. Three isonitrogenous and isocaloric complete rations, T1, T2 and T3 with 25%, 30% and 35% NDF, respectively, were fed to the experimental animals. Blood samples were collected at the beginning and the end of each of the two phases to estimate the different hematological, plasma protein, and mineral parameters to know the overall health status of the animals and standard methods were followed to analyze the samples.

**Results:**

There was no significant difference (p>0.05) in various hematological parameters such as hemoglobin, glucose, and blood urea nitrogen (BUN) in blood; plasma protein parameters such as total protein, albumin, globulin and albumin: globulin ratio and mineral parameters such as plasma calcium and phosphorus levels at the beginning and end of Phase I (1^st^ day and 90^th^ day) and Phase II (91^st^ day and 180^th^ day) as well as between the three dietary treatments, with all the values being in the normal range for lactating dairy cows. Even though nonsignificant (p>0.05), the BUN values of animals fed on ration T1, both at the beginning and end of Phase I, were higher than that of animals fed on rations T2 and T3 because the diet T1 with lowest NDF and the highest soluble carbohydrate content underwent rapid fermentation in the rumen, produced more energy, which was utilized by the rumen microbes to degrade the protein in the feed to ammonia, the excess ammonia being transported to the liver and excreted through the blood resulting in a higher BUN content.

**Conclusion:**

Feeding of paddy straw plus nonforage fiber sources based complete rations with different levels of NDF had no effect on hemato-biochemical and mineral profile as well as overall health status of lactating dairy cows. However, the higher, BUN values found in cows fed on diet T1 with 25% NDF as compared to those fed on T2 and T3 with 30% and 35% NDF, respectively, indicate more wastage of protein in T1 as compared to T2 and T3, in early lactation.

## Introduction

Energy requirements of lactating dairy cows are met from the fibrous and nonfibrous carbohydrate fractions of the diet. To achieve maximum production, dairy rations should be balanced in neutral detergent fiber (NDF), i.e., the useful fiber, at the same time not compromising on the nonfibrous carbohydrate fractions so that optimum energy intake and rumen health are ensured. The National Research Council of USA has recommended that a milking cow should be fed with a ration containing at least 25-33% of fiber in the form of NDF [1].

However, providing even this minimum quantity of fiber, from forage sources alone, is very difficult because, currently in India, green fodder is deficit to the tune of 56.73% [2]. This scarcity of green fodder could only be met in a sustained manner through the efficient use of crop residues that do not compete with human food.

Paddy and wheat straws are the principal crop residues which are used as fiber sources for ruminants in India. In the Northern states of India, wheat straw was preferred for feeding of livestock while most of the paddy straw was burnt. The need of the time was to blend straws along with locally available agro-industrial by products like brans which are nonforage fiber sources and also good sources of NDF. Once the NDF requirement is met in this manner, other concentrate ingredients can be added to it to form a complete feed or complete diet [3], which is a feasible and practical feeding practice that can be adopted. Among the various roughages such as grasses and straws, straw has got the highest rate of chewing and hence is most effective when added to complete feeds [4]. Paddy straw can be used as the sole source of forage NDF in complete rations for cows [5].

However, the optimum level of NDF in complete feeds, required to obtain maximum milk production, at the same time not compromising on its overall general health, needs to be studied. Most of the researches done on complete feeds in the western countries are grass based. In India, not much research has been conducted for formulating a paddy straw based complete feed with the optimum NDF level for lactating dairy cows.

Various hematological, plasma protein, and mineral parameters are very good indices of the overall health status of cows, in various stages of lactation [6]. However, literature available on hemato-biochemical parameters of lactating dairy cows, fed on complete rations with different levels of NDF, is scanty. Hence, this study was conducted to assess the overall health status of cows fed on paddy straw plus nonforage fiber sources based complete rations having different levels of NDF, by estimating the different hematological, plasma protein, and mineral parameters, in both early and mid lactation.

## Materials and Methods

### Ethical approval

The present study is a part of the Ph.D project (Code No. CB/1/9/DVM/2012/AN) entitled, “evaluation of complete feeds with varying levels of neutral detergent fiber for lactating dairy cows” of Dr. Biju Chacko, the first author and ethical approval for the same was provided in the 2^nd^ meeting of the Faculty Research Council of the Kerala Veterinary and Animal Sciences University, held at College of Veterinary and Animal Sciences, Mannuthy on 18-5-2013 as per Order No. DAR/Acad(2)/1769/2013(12) dt. 22-7-2013 of the Director of Academics & Research, Kerala Veterinary and Animal Sciences University, Pookode.

### Experimental design, management and laboratory analysis

A total of 18 crossbred dairy cows in the early stage of lactation (within 2 weeks of calving) were selected from the University Livestock Farm and Fodder Research and Development Scheme, Mannuthy. They were divided into three groups of six each, as uniformly as possible with regard to age, parity, milk yield, and body weight and were allotted randomly in a completely randomized design to three complete rations, T1, T2 and T3, formulated as per the recommendations of ICAR feeding standards [7]. All the cows were maintained under uniform management conditions prevailing in the farm, the average maximum and minimum temperatures being 33°C and 28°C, respectively. All the animals were dewormed with Albendazole 2.5% suspension orally, before the start of the trial. Scheduled vaccinations such as foot and mouth disease and hemorrhagic septicemia as well as standard managerial practices were followed during the experiment. The experiment was conducted from September to February.

The cows were housed individually in a well-ventilated shed with cemented floor. The animals in the three dietary treatments, T1, T2 and T3, were fed with complete rations containing different levels of NDF, viz., 25%, 30% and 35%, respectively. The rations were made isonitrogenous (12.00-13.00% crude protein) and isocaloric (63-65% total digestible nutrients) and were compared on the basis of feeding trials of total 180 days duration, in two phases of 3 months each, the first 90 days being the early lactation and the next 90 days being the mid lactation. The ingredient composition and the calculated nutrient content of the experimental rations in the two phases are depicted in Tables-1 and 2, respectively.

**Table-1:** Ingredient composition of experimental complete feeds offered to cows in Phases I and II, kg.

Ingredient	Phase I			Phase II		
	
	T1	T2	T3	T1	T2	T3
Maize	37	27	16	38	30	21
Coconut cake (de-oiled)	11	12	17	5	8	12
Rape seed meal	11	12	11	10	10	10
De-oiled rice bran	20	19	16.50	26	23	21
Paddy straw	14	21	29	14	22	29
Molasses	5	5	5	5	5	5
Calcite	1.50	1.50	1.50	1.5	1.5	1.5
Salt	0.50	0.50	0.50	0.5	0.5	0.5
Vegetable fat	-	2.00	3.50	-	-	-
Total	100*	100*	100*	100*	100*	100*

* To every 1 kg of complete feed, 0.10 g of vitamin AD_3_E supplement (containing 10,000 IU of vitamin A, 2000 IU of vitamin D_3_ and 1000 IU of vitamin E), 0.50 g of trace mineral mixture (Keramin Forte) and 0.50 g of toxin binder (Curatox) were added

**Table-2:** Chemical composition of complete offered to cows in Phases I and II (% DM basis).

Nutrient	Phase I			Phase II		
	
	T1	T2	T3	T1	T2	T3
CP	12.23	12.94	12.18	12.40	12.14	12.08
Crude fiber	10.73	12.68	15.01	10.53	12.70	14.85
Ether extract	4.00	3.60	3.80	4.50	3.50	3.60
Total ash	14.00	12.90	12.90	14.10	13.30	12.98
Nitrogen free extract	59.04	57.88	56.11	58.47	58.36	56.49
Acid insoluble ash	5.30	5.60	6.00	4.80	5.40	6.20
NDF	25.88	30.03	35.59	25.94	30.86	35.38
ADF	21.60	23.10	24.80	21.80	23.00	25.01
Calcium	0.83	0.85	0.87	0.81	0.84	0.86
Phosphorus	0.54	0.52	0.48	0.59	0.55	0.52
TDN*	64.50	64.44	64.28	64.04	63.28	63.25
Metabolisable energy* (MJ/kg DM)	9.00	8.87	8.41	9.04	8.54	8.24

* Calculated value.

ADF=Acid detergent fiber, NDF=Neutral detergent fiber, TDN=Total digestible nutrients, CP=Crude protein, DM=Dry matter

Weighed quantities of complete feed were fed individually, on an *ad libitum* basis, to all the animals and the balance feed in the manger was collected manually and weighed, twice a day, in the morning and afternoon at 9 AM and 2 PM, respectively. *Ad libitum* drinking water was provided to the cows using automatic watering system. Proximate principles of feed were determined as per standard procedure of AOAC [8]. The acid detergent fiber and NDF were estimated by the detergent method of analysis [9]. The calcium and phosphorus contents of the feed were analyzed as per the standard procedure described in AOAC [8].

### Estimation of hemato-biochemical and mineral parameters

Blood samples (15 ml each) were collected aseptically by puncturing the jugular vein using 18 gauge disposable needle into two sets of sterilized vials, one without anticoagulant and the other with anticoagulant (1^st^ day), at the end of Phase I (90^th^ day) and at the end of the experiment (180^th^ day), before the morning feeding. Blood samples with ethylenediaminetetraacetic acid were used immediately for estimation of hemoglobin (cyanmethemoglobin method) using the standard kits supplied by Agappe Diagnostics, Maharashtra, India.

Blood without anticoagulant was allowed to clot; the plasma was separated in a fresh vial and stored at −20°C for the analysis of blood glucose (glycerol-3-phosphate oxidase-phenol + aminophenazone method), blood urea nitrogen (BUN) (modified Berthelot method), total protein (direct Biuret method), albumin (bromocresol green methodology), calcium (modified o-cresolphthalein complexone method), and inorganic phosphorus (phosphomolybdate method) using the standard kits supplied by Agappe Diagnostics to assess the overall health status of cows fed on paddy straw plus nonforage fiber sources based complete rations having different levels of NDF. All the hematological, plasma protein, and mineral parameters listed above were determined using the Autoanalyzer (Mispa plus, SEAC radium group). Plasma globulin was calculated by subtracting albumin from total protein and albumin: globulin ratio was calculated using the formula: Albumin÷globulin.

### Statistical analysis

Data gathered on the various parameters were analyzed statistically using single factor analysis of variance technique [10], using the software, statistical product and service solutions (SPSS) version 21.0 [11]. Homogenous subsets were separated using Duncan’s multiple range test, described by Duncan [12]. Differences among treatments were considered to be significant, when p≤0.05.

## Results

In this study different hematological, plasma protein and mineral parameters, in both early and mid lactation, were observed.

### Hematological parameters

The data on the values of blood parameters such as hemoglobin, glucose, and BUN of blood samples collected from the experimental animals in Phases I and II of the experiment are given in Table-3.

**Table-3:** Blood*, plasma protein* and mineral parameters* of animals maintained on the three experimental rations in Phases I and II.

Parameter	Day	Treatment			p value

		T1	T2	T3	
Hemoglobin (g/100 ml)	1	10.45±0.50	10.76±0.72	10.78±0.87	0.934
	90	9.72±0.37	10.53±0.52	10.49±0.76	0.543
	180	9.89±0.39	11.31±0.60	11.20±0.86	0.253
Blood glucose (mg/100 ml)	1	67.07±1.57	66.19±1.23	66.54±0.62	0.874
	90	62.44±0.91	63.65±1.23	62.60±0.58	0.625
	180	66.11±1.09	66.10±0.78	65.94±0.50	0.987
BUN (mg/100 ml)	1	14.05±1.09	12.97±0.33	12.95±0.52	0.486
	90	11.23±1.25	9.64±0.49	9.86±0.51	0.366
	180	7.82±0.45	8.49±0.24	8.64±0.31	0.230
Total protein (g/100 ml)	1	7.31±0.33	7.47±0.16	7.17±0.3	0.749
	90	7.06±0.35	7.00±0.12	7.14±0.33	0.941
	180	7.38±0.34	7.52±0.15	7.58±0.24	0.845
Albumin (g/100 ml)	1	4.18±0.14	4.17±0.09	4.17±0.2	0.998
	90	3.96±0.20	3.95±0.17	3.94±0.24	0.996
	180	4.26±0.18	4.27±0.20	4.24±0.19	0.992
Globulin (g/100 ml)	1	3.13±0.22	3.31±0.08	3.00±0.19	0.487
	90	3.10±0.15	3.05±0.06	3.21±0.21	0.762
	180	3.12±0.16	3.25±0.32	3.35±0.12	0.764
Albumin: globulin ratio	1	1.36±0.09	1.26±0.02	1.41±0.11	0.433
	90	1.28±0.01	1.30±0.08	1.25±0.11	0.902
	180	1.37±0.02	1.41±0.20	1.27±0.07	0.726
Plasma Ca (mg/100 ml)	1	9.11±0.49	9.33±0.49	8.83±0.46	0.766
	90	8.23±0.07	8.41±0.33	8.15±0.25	0.754
	180	9.68±0.46	9.87±0.76	9.43±0.24	0.848
Plasma P (mg/100 ml)	1	4.41±0.07	4.83±0.21	4.72±0.17	0.199
	90	5.03±0.13	5.23±0.25	5.26±0.32	0.772
	180	5.37±0.20	5.47±0.33	5.69±0.29	0.711

* Average of six values. BUN=Blood urea nitrogen

The average hemoglobin levels in animals fed on the three experimental rations T1, T2 and T3, at the beginning of the experiment (1^st^ day) were 10.45±0.50, 10.76±0.72 and 10.78±0.87 g/100 ml, respectively. The values at the end of Phase I (90^th^ day) were 9.72±0.37, 10.53±0.52 and 10.49±0.76 g/100 ml, respectively, for animals fed on T1, T2 and T3, and the values at the end of Phase II (180^th^ day) were 9.89±0.39, 11.31±0.60 and 11.20±0.86 g/100 ml, respectively, for cows fed on T1, T2 and T3. The above data revealed that there was no significant difference (p>0.05) in hemoglobin values between the three dietary treatments, throughout Phases I and II and the values were in the normal range for cows.

The initial values (1^st^ day) of average blood glucose levels of animals fed on the three experimental rations, T1, T2 and T3 were 67.07±1.57, 66.19±1.23 and 66.54±0.62 mg/100 ml, respectively. The values at the end of Phase I (90^th^ day) were 62.44±0.91, 63.65±1.23 and 62.60±0.58 mg/100 ml for the animals in T1, T2 and T3, respectively, and the values at the end of Phase II (180^th^ day) were 66.11±1.09, 66.10±0.78 and 65.94±0.50 mg/100 ml, respectively, for cows fed on T1, T2 and T3. The above data revealed that there was no significant difference (p>0.05) in blood glucose levels of cows fed on the three experimental rations, throughout Phase I and Phase II and the values were in the normal range for lactating cows.

The BUN concentration of animals fed on the three experimental rations; T1, T2 and T3 were 14.05±1.09, 12.97±0.33 and 12.95±0.52 mg/100 ml, respectively, at the start of the experiment (1^st^ day). The BUN values at the end of Phase I (90^th^ day) were 11.23±1.25, 9.64±0.49 and 9.86±0.51 mg/100 ml, respectively, for the animals fed on the three experimental rations, T1, T2 and T3. The BUN values at the end of Phase II (180^th^ day) were 7.82±0.45, 8.49±0.24 and 8.64±0.31 mg/100 ml, respectively, for the animals fed on T1, T2 and T3. The above data indicate that there was no significant difference (p>0.05) in BUN values between the three dietary treatments, throughout Phases I and II and the values were in the normal range for lactating cows.

### Plasma protein parameters

The data on values of plasma protein parameters such as total protein, albumin, globulin and albumin:globulin ratio, in Phases I and II are depicted in Table-3.

The average total protein content in animals fed on the three experimental rations T1, T2 and T3; at the beginning of the experiment (1^st^ day) were 7.31±0.33, 7.47±0.16 and 7.17±0.30 g/100 ml, respectively. The values at the end of Phase I (90^th^ day) were 7.06±0.35, 7.00±0.12 and 7.14±0.33 g/100 ml, respectively, for the animals fed on T1, T2 and T3 and the values at the end of Phase II (180^th^ day) were 7.38±0.34, 7.52±0.15 and 7.58±0.24 g/100 ml, respectively, for the animals fed on T1, T2 and T3. The above data revealed that there was no significant difference (p>0.05) in total protein levels between the three dietary treatments, throughout Phase I and Phase II and the values were in the normal range for cows.

The average albumin content in animals fed on the three experimental rations T1, T2 and T3, at the beginning of the experiment (1^st^ day) were 4.18±0.14, 4.17±0.09 and 4.17±0.20 g/100 ml, respectively. The values at the end of Phase I (90^th^ day) were 3.96±0.20, 3.95±0.17 and 3.94±0.24 g/100 ml, respectively, for the animals fed on T1, T2 and T3, and the values at the end of Phase II (180^th^ day) were 4.26±0.18, 4.27±0.20 and 4.24±0.19 g/100 ml, respectively, for cows fed on T1, T2 and T3. The above data revealed that there was no significant difference (p>0.05) in albumin levels between the three dietary treatments, throughout Phase I and Phase II and the values were in the normal range for cows.

The average globulin content in animals fed on the three experimental rations T1, T2 and T3, at the beginning of the experiment (1^st^ day) was 3.13±0.22, 3.31±0.08 and 3.00±0.19 g/100 ml, respectively. The values at the end of Phase I (90^th^ day) were 3.10±0.15, 3.05±0.06 and 3.21±0.21 g/100 ml, respectively, for the animals fed on T1, T2 and T3 and the values at the end of Phase II (180^th^ day) were 3.12±0.16, 3.25±0.32 and 3.35±0.12 g/100 ml, respectively, for cows fed on T1, T2 and T3. The above data revealed that there was no significant difference (p>0.05) in globulin levels between the three dietary treatments, throughout Phases I and II and the values were in the normal range for cows.

The albumin:globulin ratio in animals fed on the three experimental rations T1, T2 and T3, at the beginning of the experiment (1^st^ day) was 1.36±0.09, 1.26±0.02 and 1.41±0.11, respectively. The values at the end of Phase I (90^th^ day) was 1.28±0.01, 1.30 ±0.08 and 1.25±0.11, respectively, for the animals fed on T1, T2 and T3 and the values at the end of Phase II (180^th^ day) were 1.37±0.02, 1.41±0.20 and 1.27±0.07, respectively, for cows fed on T1, T2 and T3. The above data revealed that there was no significant difference (p>0.05) in albumin: Globulin ratio between the three dietary treatments, throughout Phases I and II and the values were within the normal range for cattle.

### Mineral parameters

The data on values of mineral parameters such as plasma calcium and phosphorus, in Phase I and II are depicted in Table-3.

The plasma calcium content in animals fed on the three experimental rations T1, T2 and T3, at the beginning of the experiment (1^st^ day) was 9.11±0.49, 9.33±0.49 and 8.83±0.46 mg/100 ml, respectively. The values at the end of Phase I (90^th^ day) were 8.23±0.07, 8.41±0.33 and 8.15±0.25 mg/100 ml, respectively, for cows fed on T1, T2 and T3 and the values at the end of Phase II (180^th^ day) were 9.68±0.46, 9.87±0.76 and 9.43±0.24 mg/100 ml, respectively, for those fed on T1, T2 and T3. The above data revealed that there was no significant difference (p>0.05) in plasma calcium content between the three dietary treatments, throughout Phase I and the values were within the normal range for dairy cows.

The plasma phosphorus content in animals fed on the three experimental rations T1, T2 and T3, at the beginning of the experiment (1^st^ day) were 4.41±0.07, 4.83±0.21 and 4.72±0.17 mg/100 ml, respectively. The values at the end of Phase I (90^th^ day) were 5.03±0.13, 5.23±0.25 and 5.26±0.32 mg/100 ml, respectively, for the animals fed on T1, T2 and T3. The values at the end of Phase II were 5.37±0.20, 5.47±0.33 and 5.69±0.29 mg/100 ml, respectively, for the cows fed on T1, T2 and T3. The above data revealed that there was no significant difference (p>0.05) in plasma phosphorus content between the three dietary treatments, throughout Phase I and the values were within the normal range for dairy cows.

## Discussion

### Hematological parameters

The hemoglobin values obtained in Phases I and II of the present study are comparable to those of Ally [13] who reported values in the range of 8.35-10.70 g/100 ml in early lactation and 9.80-11.00 g/100 ml in mid-lactation dairy cows fed on conventional paddy straw-concentrate rations where the NDF content of the total ration was in the range of 30.72-34.76% and 38.65-39.40% in early and mid-lactation, respectively. The values obtained in the present study are also comparable to the values of Mohan [14] who reported hemoglobin values in the range of 8.61-9.75 g/100 ml and 9.49-11.25 g/100 ml, respectively, in dairy cows fed on conventional paddy straw-concentrate rations with total NDF content of 38.62% and 43.55%, respectively, in the 3^rd^ and 6^th^ month of lactation. The values of the current study are higher than those reported by Mondal and Paul [15] who obtained hemoglobin values in the range of 9.13-9.64 g/100 ml in lactating multiparous dairy cows.

The blood glucose values obtained in Phases I and II of the present study are higher than those reported by Ally [13] who reported plasma glucose values in the range of 51-62 mg/100 ml early lactation and 43.57-62.14 mg/100 ml in mid-lactation dairy cows fed on conventional paddy straw-concentrate rations; Delahoy *et al*. [16] who reported blood glucose content of 69.20 and 68.50 mg/100 ml, respectively, in cows fed on complete rations containing cracked and steam flaked corn; Hundal *et al*. [17] who reported values in the range of 54.46-54.98 mg/100 ml in lactating cows fed on total mixed rations (TMRs); Shiraz *et al*. [18] who got plasma glucose values in the range of 56.56-64.58 mg/100 ml in crossbred cows fed on wheat straw based TMRs; Mondal and Paul [15] who got blood glucose values in the range of 42.40-49.20 mg/100 ml, in lactating multiparous dairy cows and Shakkarpude *et al*. [19] who obtained values in the range of 61.33-64.50 mg/100 ml in lactating crossbred dairy cows. The values obtained in are in the present study are lower than the values of Kim *et al*. [20] who reported that the blood glucose content of Hanwoo steers fed on TMRs with and without fermented feed were in the range of 86.50-91.50 mg/100 ml.

The BUN values observed in the present study are comparable to those reported by Hundal *et al*. [17] who observed that crossbred milking cows fed on a TMR had a BUN value of 13.00 mg/100 ml. The BUN values of the present investigation are also comparable to those of Kim *et al*. [20] who reported that the BUN content of Hanwoo steers fed on TMRs, with and without fermented feed, ranged from 9.82 to 13.30 mg/100 ml. The values obtained in Phases I and II of the present study are lower than those reported by Ally [13] who reported BUN values in the range of 13.60-21.50 mg/100 ml in early lactation and 35.30-44.40 mg/100 ml in mid-lactation dairy cows fed on conventional paddy straw-concentrate rations; Delahoy *et al*. [16] who obtained plasma urea nitrogen values of 12.50 and 13.70 mg/100 ml in lactating dairy cows fed on complete rations containing steam-flaked and cracked corn, respectively and Pina *et al*. [21] who reported that the urea nitrogen concentration of serum in lactating Holstein cows fed on complete diets consisting of four different protein sources were in the range of 18.24-22.97 mg/100 ml.

Perusal of the data on BUN of the experimental animals fed on the three experimental rations, given in Table-3 revealed that the BUN values of animals in T1, both at the beginning and end of Phase I, were higher than that of animals in T2 and T3, even though the increases were non-significant (p>0.05). The higher BUN values observed in T1 was because the diet T1 with lowest NDF and the highest soluble carbohydrate content underwent rapid fermentation in the rumen, and the resultant energy produced was utilized by the rumen microbes to degrade the protein in the feed to ammonia which in turn resulted in an increase in rumen ammonia. The excess ammonia produced was transported to the liver and excreted through the blood which resulted in a higher BUN content in T1 than T2 and T3, which indicated more wastage of protein in T1 as compared to T2 and T3, in early lactation.

### Plasma protein parameters

The values of total protein obtained in this study are comparable to those of Shakkarpude *et al*. [19] who got values in the range of 7.25-7.97 g/100 ml in lactating crossbred dairy cows. The values observed in the present study are higher than those of Shiraz *et al*. [18] who reported plasma total protein values in the range of 6.62-6.97 g/100 ml in crossbred milch cows in early lactation, fed on wheat straw based TMRs. The values are also higher than those observed by Kim *et al*. [20] who reported that the total protein content of Hanwoo steers fed on a TMR with and without fermented feed were in the range of 6.11-6.28 g/100 ml, and Mondal and Paul [15] who reported plasma total protein values in the range of 6.62-6.97 g/100 ml in lactating multiparous dairy cows. The values obtained in this experiment are lower than that reported by Hundal *et al*. [17] who reported that the total protein content in crossbred milking cows fed on a TMR was 9.08 g/100 ml.

The values of albumin obtained in this study are comparable to those reported by Hundal *et al*. [17] who observed that the albumin content in plasma of crossbred milking cows fed on a TMR was 3.52 and 4.76 g/100 ml. The values of albumin observed in the present study are higher than those of Shiraz *et al*. [18] who reported plasma albumin values in the range of 2.95-3.18 g/100 ml in crossbred milch cows fed on wheat straw-based TMRs; Kim *et al*. [20] who reported that the total protein content of Hanwoo steers fed on a TMR with and without fermented feed was in the range of 3.65-3.89 g/100 ml and Mondal and Paul [15] who reported plasma albumin values in the range of 2.71-2.77 g/100 ml in lactating multiparous dairy cows.

The values of globulin reported in the present study are higher than those observed by Mondal and Paul [15] who reported plasma globulin values in the range of 2.82-2.89 g/100 ml in lactating multiparous dairy cows. The values of globulin obtained in this study are lower than those reported by Hundal *et al*. [17] who observed that the globulin content in plasma of crossbred milking cows fed on a TMR was 5.57 g/100 ml, and Shiraz *et al*. [18] who got plasma globulin values in the range of 3.58-3.82 g/100 ml in crossbred milch cows fed on wheat straw based TMRs.

The values of albumin:globulin ratio obtained in this study are comparable to those of Raghuvansi *et al*. [22] who reported an albumin:globulin ratio of 1.03-1.54 in Malpura rams fed on pearl millet stover based complete feeds and higher than those observed by Mondal and Paul [15] who reported values in the range of 0.96-0.98 in lactating multiparous dairy cows.

### Mineral parameters

The values of plasma calcium observed in this study are comparable to those of Ally [13] who reported values ranging from 8.33 to 9.67 mg/100 ml in a study conducted to assess the effect of varying levels of dietary protein as well as varying degradability of the protein in lactating dairy cows fed on conventional paddy straw-concentrate rations. The plasma calcium values of this study are also comparable to those observed by Cozzi *et al*. [23] who analyzed the blood samples of 740 Holstein cows collected from 33 Italian dairy herds and reported that the mean serum calcium was 9.62 mg/100 ml and Dominic [24] who conducted a study to find out the effect of high energy diet in early lactation and reported values in the range of 9.01-9.91 mg/100 ml in lactating dairy cows. The calcium values reported in this study are higher than those of Jacob [25] who got values ranging from 7.30 to 7.47 mg/100 ml in a study conducted to assess the mineral status during pregnancy in crossbred cattle. The plasma calcium values of this study are lower than those of Mohan [14] who reported values ranging from 10.00 to 11.25/100 ml in a study conducted to assess the effect of dietary level of minerals in lactating crossbred dairy cows.

The values of plasma phosphorus observed in the present study are comparable to those of Shakkarpude *et al*. [19] who conducted studies to assess the effect of plasma phosphorus on reproductive performance and reported inorganic phosphorus values in the range of 4.40-5.43 mg/100 ml in lactating crossbred dairy cows. The values are also comparable to those of Dominic [24] who reported plasma phosphorus values in the range of 4.78-5.04 mg/100 ml in lactating dairy cows. The values of plasma phosphorus observed in the present study were lower than those of Jacob [25] who reported plasma phosphorus values ranging from 6.02 to 6.20 mg/100 ml; Ally [13] who reported values ranging from 6.24 to 7.74 mg/100 ml; Mohan [14] who obtained values ranging from 6.01 to 6.40 mg/100 ml in lactating crossbred cows and Cozzi *et al*. [23] who reported that the mean value of serum phosphorus was 6.57 mg/100 ml in Holstein cows.

Normal blood levels of various biochemical constituents are indispensable for normal functioning of various systems of the body [26]. The normal values of all blood, plasma protein, and mineral parameters of cows fed on the three experimental rations, T1, T2 and T3, in Phases I and II, observed in the present study must have contributed to the overall health status of the animals in all the dietary treatments.

## Conclusion

Feeding of paddy straw plus nonforage fiber sources based complete rations with different levels of NDF had no effect on hemato-biochemical and mineral profile as well as overall health status of lactating dairy cows. However, the higher, BUN values found in cows fed on diet T1 with 25% NDF as compared to those fed on T2 and T3 with 30% and 35% NDF, respectively, indicate more wastage of protein in T1 as compared to T2 and T3, in early lactation. The results of the present investigation reveal that complete rations with 25-35% NDF, containing paddy straw as the sole source of roughage NDF, can be recommended for use among early and mid-lactation dairy cows, with 30% and 35% being the ideal NDF level.

## Authors’ Contributions

BC, KMSM, KA, and KS designed all steps of the study. BC, KSA, and CTS collected samples of blood and evaluated throughout the study. BC and KMSM analyzed the data statistically. BC, KMSM, KA and KS prepared the tables of results and discussed it. BC wrote the manuscript draft and all authors have read, revised and approved the final draft.
